# Written Exposure Therapy as Transformative, Scalable Care for Posttraumatic Stress Disorder

**DOI:** 10.2196/103501

**Published:** 2026-06-15

**Authors:** Candice Marie Sage

**Keywords:** posttraumatic stress disorder, mental health services, global health, access to health care, scalability, psychological intervention, narrative medicine, digital narrative medicine

## Abstract

International organizations are calling for urgent scaling of mental health care services, with the World Health Organization noting over a billion people living with mental health conditions in 2025. In this *News and Perspectives* article, JMIR Correspondent Candice Marie Sage reports on a brief scalable intervention for posttraumatic stress disorder.

**Key Takeaways:**

- Written exposure therapy (WET) is a brief intervention for posttraumatic stress disorder that shows promise compared to gold-standard treatments.
- WET shows high uptake, feasibility, and scalability in real-world care settings.
- WET can promote health equity through online deployment as a digital narrative medicine intervention.

Candice Marie Sage, PhD, is a researcher with over 20 years of experience managing interdisciplinary research in Canada and internationally. She is an expert in knowledge cocreation, translation, and exchange. In this piece, she continues her series on digital narrative medicine stories and applications.

Posttraumatic stress disorder (PTSD) impacts 3.9% of the population worldwide, with significant psychiatric and medical sickness in the absence of effective treatment. According to World Health Organization data across 15 countries, only 43.0% of people diagnosed with PTSD over a 12-month period were able to access mental health services. Of those, only 13.5% received adequate pharmacotherapy and 17.2% adequate psychotherapy. Accessibility drops for populations such as veterans and those without insurance, as well as low- and middle-income countries.

As a person with complex PTSD in the Canadian context, it has taken me over two years to access publicly funded care, and my healing journey is far from over. Affordability as well as a shortage of qualified mental health professionals are barriers to PTSD care in Canada.

What if one of the most powerful interventions to address PTSD begins with a story written by the patients themselves? An evidence-based PTSD treatment known as written exposure therapy (WET) asks patients to write the story of their traumatic experience across five facilitated, structured sessions. These patient-authored narratives—including in-person and telehealth digital formats within digital narrative medicine—can help bridge the gap to accessing effective PTSD treatment. WET shows promise as a scalable, much needed public health intervention.

## As Effective as More Intensive PTSD Treatments

The WET process was codeveloped by Denise Sloan, PhD, a clinical psychologist whose research focuses on narrative processing, and Brian Marx, PhD, a clinician investigator studying the implementation of trauma therapies in health care systems. Working from the US Department of Veterans Affairs’ National Center for PTSD, their collaboration began in 2004, investigating expressive writing as a treatment for PTSD symptoms. The WET protocol was refined over a decade of systematic empirical studies. In 2019, a treatment manual for WET was widely disseminated as a clearly defined clinical approach. A 2024 systematic review that examined a variety of settings and trauma populations across several countries found WET to be an efficacious and effective treatment for PTSD symptoms.

While WET is often delivered in person in clinical settings, it can also be delivered via video meetings, with patients submitting images of their written accounts via a secure and confidential electronic system. As piloted during the COVID-19 lockdown, digitally delivered WET supported feasibility and flexibility in different settings with similar effectiveness as in-person care.

I interviewed a WET practitioner and trainer, Stefanie T LoSavio, PhD, ABPP, associate professor of psychiatry and behavioral sciences and director of research and innovation at the STRONG STAR Training Initiative at the University of Texas at San Antonio. LoSavio emphasized that WET has shown clinically significant reductions in PTSD symptoms and is comparable in effectiveness to more intensive treatments like cognitive processing therapy and prolonged exposure therapy, despite being much shorter. WET’s brevity and reduced therapist interaction were key innovations when it first gained attention in the field.

## Activating Patients’ Natural Recovery Mechanisms

Inquiring how WET works so well with less therapist interaction, LoSavio credited the narrative writing process itself. WET involves confronting the memory of a traumatic event so that the person can process and recover from the experience. They do this by writing repeatedly about the details of the event and, later, its impact on them. By approaching and processing the memory instead of avoiding it, the person can learn that they can tolerate the memory, lessen distress associated with it, and make sense of the experience such that it becomes a part of their history instead of showing up in daily PTSD symptoms. Patients often experience cognitive shifts spontaneously through writing about their trauma, which helps them remember details and address distressing beliefs like why the event happened and why it was not prevented.

**Figure:**
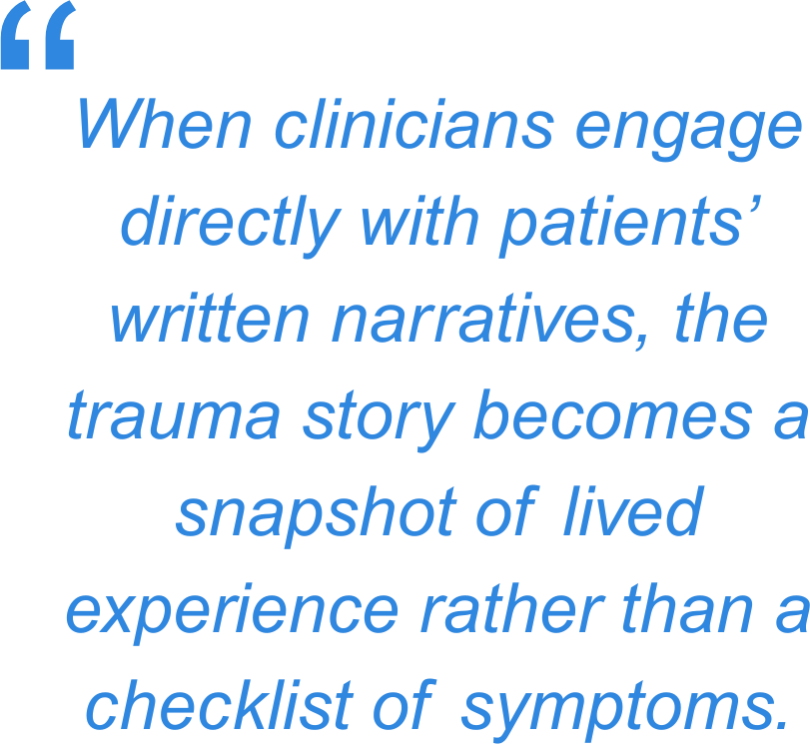


LoSavio notes, “Patients have an opportunity to recall the full details of the trauma, including what happened and what they were thinking and feeling at the time. They’re not writing it like a report for someone else but for themselves in order to recover from the trauma.”

Progress occurs as patients access their natural recovery mechanisms by addressing avoidance and enabling the processing of traumatic memories. She says, “People have good mechanisms to process and recover from bad events, but patients with PTSD get ‘stuck’ in the process.” Interestingly, LoSavio notes that some patients continue to improve even after the treatment ends, carrying on their own processing work past the therapeutic sessions.

She reports that patients typically find the writing process gets easier over time as they develop a sense of increased control over their memories rather than being controlled by them. Their confidence grows in their competence to feel their emotions rather than avoiding them. Also, for many, recounting the details of the trauma in their writing can help them reconsider unhelpful interpretations, like erroneously assuming their trauma was their fault.

Patient narratives can become more than a therapeutic exercise: they can function as a bridge between patient experience and clinical care. When clinicians engage directly with patients’ written narratives, the trauma story becomes a snapshot of lived experience rather than a checklist of symptoms. This could deepen empathy, improve diagnostic clarity, and help clinicians understand how trauma shapes a patient’s life beyond the clinical encounter.

LoSavio noted that while some clinicians have delivered WET in a group format, published clinical trials involve individual therapy. She trains providers in WET through foundational workshops and consultation calls. Therapists can seek guidance on patient appropriateness, treatment planning, and feedback on deidentified patient narratives. The next frontier of training involves professionals like peer supports whose roles intersect with patients’ lives. This can alleviate wait times to access WET where there is a shortage of therapists and could be part of a larger public health strategy. She detailed the ethical considerations around handling patient narratives, including secure storage and disposal methods, emphasizing the importance of maintaining patient privacy. Thus, narratives are not meant to be stored in electronic patient records or student files.

## Digital Delivery to Scale WET to Underserved Populations

LoSavio is confident that the development and empirical basis of WET holds potential for broader implementation of this brief effective treatment. Further research will inform culturally responsive care and alternative delivery methods. Decentralization of care and integration of WET into existing nonspecialty settings is happening in the United States, with treatment delivered in primary care clinics, addiction programs, and veterans’ telehealth systems, and to people experiencing homelessness. She notes that studies show comparable outcomes in telehealth versus in-person program delivery, with the potential to reach rural populations, mobility-limited patients, and underserved communities by expanding reach beyond traditional clinics.

WET is a pathway to effective and more accessible PTSD care. Technology as a scaling tool can further foster health equity by reaching the kinds of populations often excluded from traditional trauma therapy due to cost or specialist bottlenecks.

“PTSD is treatable, and it doesn’t have to take years,” LoSavio concludes. “Current reach of evidence-based treatments for PTSD is low, and WET can help with access.”

Indeed, WET is a promising example of interventions and technology that could alleviate much human suffering.

